# Association of maternal hypertensive disorders in pregnancy with infant neurodevelopment

**DOI:** 10.7555/JBR.37.20230074

**Published:** 2023-09-28

**Authors:** Bo Huang, Yifan Wang, Yangqian Jiang, Hong Lv, Tao Jiang, Yun Qiu, Qun Lu, Jiangbo Du, Yuan Lin, Hongxia Ma

**Affiliations:** 1 State Key Laboratory of Reproductive Medicine, Nanjing Medical University, Nanjing, Jiangsu 211166, China; 2 Department of Epidemiology, Center for Global Health, School of Public Health, Nanjing Medical University, Nanjing, Jiangsu 211166, China; 3 Department of Maternal, Child and Adolescent Health, Center for Global Health, School of Public Health, Nanjing Medical University, Nanjing, Jiangsu 211166, China; 4 State Key Laboratory of Reproductive Medicine (Suzhou Centre), the Affiliated Suzhou Hospital of Nanjing Medical University, Suzhou Municipal Hospital, Gusu School, Nanjing Medical University, Suzhou, Jiangsu 215002, China; 5 Department of Biostatistics, School of Public Health, Nanjing Medical University, Nanjing, Jiangsu 211166, China

**Keywords:** hypertensive disorders in pregnancy, infant neurodevelopment, prospective birth cohort study

## Abstract

Inconsistent findings have been reported regarding the associations between hypertensive disorders in pregnancy (HDP) and infant neurodevelopment. Leveraging data from the Jiangsu Birth Cohort, in the present study, we re-visited such associations in one-year-old infants from 2576 singleton pregnancies and 261 twin pregnancies. We first assessed infant neurodevelopment by the Bayley Scales of Infant and Toddler Development Screening Test (the Third Edition), and then estimated its association with maternal HDP using general linear regression models and Poisson regression models. In singleton pregnancies, compared with mothers unexposed to HDP, infants born to mothers with chronic hypertension exhibited a lower score ( *β*, −0.67; 95% confidence interval [CI], −1.19–−0.15) and a higher risk of "non-optimal" gross motor development (risk ratio [RR], 2.21; 95% CI, 1.02–4.79); in twin pregnancies, infants born to mothers with HDP exhibited lower scores in cognition ( *β*, −0.49; 95% CI, −0.96–−0.01), receptive communication ( *β*, −0.55; 95% CI, −1.03–−0.06), and gross motor ( *β*, −0.44; 95% CI, −0.86–−0.03), and at a higher risk of "non-optimal" gross motor development (RR, 2.12; 95% CI, 1.16–3.88). These findings indicate that infants born to mothers with HDP may have inferior neurodevelopment outcomes at the age of one year.

## Introduction

Hypertensive disorders in pregnancy (HDP) encompass a range of conditions, including chronic hypertension, gestational hypertension, preeclampsia, and preeclampsia superimposed on chronic hypertension ^[ 1] ^. These conditions collectively affect up to 10% of pregnancies worldwide ^[ 2] ^. A number of studies have provided evidence for the effect of HDP on the development of perinatal infants, such as premature delivery, low birth weight, and fetal respiratory distress ^[ 3– 4] ^. Besides, emerging data indicate that maternal HDP may have long-term effects on child development and health ^[ 5] ^.

The fetal brain is highly plastic and susceptible to an adverse intrauterine environment ^[ 6] ^. Prenatal exposure to HDP may lead to fetal intrauterine hypoxia, confering critical long-term consequences for future child neurodevelopment ^[ 7] ^. Based on the registries on birth, disease, and hospitalization, previous studies have reported the associations between HDP and serious neurodevelopmental diseases, including autism spectrum disorders, developmental delay, and intellectual disability ^[ 8– 9] ^. In addition to clinical diagnoses, a series of studies have focused on the associations between HDP and child neurodevelopment. However, the results are not consistent. For example, studies have reported a mix of negative ^[ 10– 12] ^, positive ^[ 13– 14] ^, or null ^[ 15– 16]^ effects of HDP on infant neurodevelopment. Moreover, previous studies also reported inconsistent effects of HDP on the different dimensions of neurodevelopment. For example, the Northern Finland Birth Cohort followed up 8847 singleton children for up to 11.5 years, and found that maternal gestational hypertension increased the risk of mild cognitive impairment in the offspring ^[ 17] ^. However, another study found that HDP was a risk factor for a slight reduction in the verbal ability of offsprings at 10 years old, but no significant association was found between HDP and cognition ability ^[ 18] ^. Therefore, more studies are needed to investigate the associations between maternal HDP exposure and infant neurodevelopmental outcomes.

In the present study, we examined the data from a perspective cohort to investigate the impact of maternal HDP on the neurodevelopment of infants at one year old. We evaluated whether infants born to mothers with HDP had lower scores of neurodevelopment and higher risks of "non-optimal" neurodevelopment in cognitive, receptive communication, expressive communication, fine motor, and gross motor domains.

## Subjects and methods

### Study design and study population

**Figure 1:**
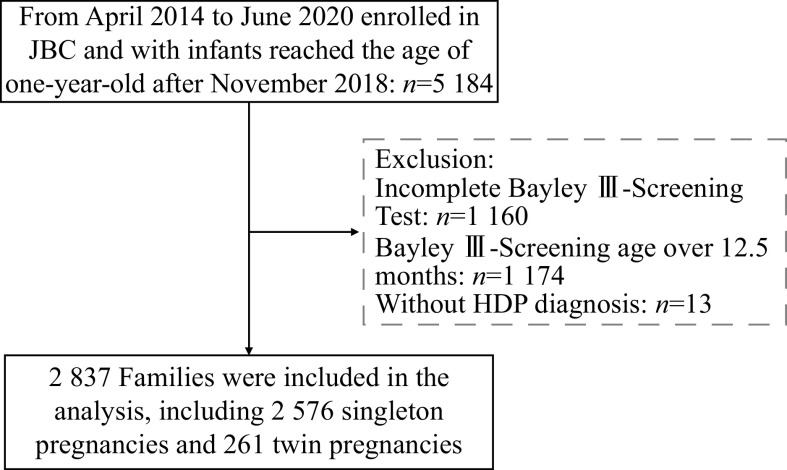
Flowchart of participants throughout the study.

The present study used the resources from a prospective cohort study, the Jiangsu Birth Cohort (JBC). Details of the JBC study have been published elsewhere ^[ 19] ^, and this cohort study had recruited and followed up the couples with spontaneous conception or assisted reproductive technology (ART) conception in Jiangsu, China, between April 2014 and June 2020, and systematically evaluated the short- and long-term health outcomes and well-being of ART-births. Since November 2018, the Bayley Scales of Infant and Toddler Development Screening Test (Third Edition, Bayley Ⅲ-Screening Test) has been used to assess infant neurodevelopment at one-year-old follow-up in the JBC. The present study included families from the Women's Hospital of Nanjing Medical University (Nanjing) and the Suzhou Affiliated Hospital of Nanjing Medical University (Suzhou) before June 30, 2020, and infants who had reached the age of one year old after November 2018 were selected. Of the 5184 families, 2850 families had completed neurodevelopmental assessments between the age of 11–12.5 months, and 13 families without HDP diagnosis information were excluded. As a result, 2837 families were included in the present study, including2576 singleton pregnancies and 261 twin pregnancies. The flowchart of participants recruited for the present study is shown in ***Fig. 1***. The Human Research Ethics Committee of Nanjing Medical University approved all study procedures (NJMU-2016-311). A written informed consent was obtained from all participants, and the present study was conducted following the Declaration of Helsinki.

### Definition of maternal hypertensive disorders in pregnancy

HDP is a group of diseases, including chronic hypertension, gestational hypertension, preeclampsia, and preeclampsia superimposed on chronic hypertension ^[ 1] ^. According to the diagnostic criteria of American College of Obstetricians and Gynecologists, maternal HDP diagnosis was confirmed ^[ 20] ^. In the study, we merged preeclampsia superimposed on the chronic hypertension group into the chronic hypertension group. We determined maternal HDP based on relevant test data recorded in the medical information system.

### Assessment of infant neurodevelopment

Occupational therapists or pediatricians with standardized training used the Bayley-Ⅲ Screening Test to evaluate the neurodevelopment of one-year-old infants in the presence of their primary caregivers. The Bayley-Ⅲ Screening Test ^[ 21]^ served as a comprehensive assessment tool for the neurodevelopment of infants from one to 42 months old, which consists of five domains: cognitive, receptive communication, expressive communication, fine motor, and gross motor scales. Each domain has a series of developmental playing tasks, and one point will be given after completing a task. The higher the score, the better the development. According to the previously determined critical points, each subtest can be divided into three categories: at-risk, emerging, and competent. The detailed classification standards are shown in ***Supplementary Table 1*** (available online). Considering the number of "at-risk" infants was small, we combined the two categories of "emerging" and "at-risk" as "non-optimal" in neurodevelopment. JBC adopted a series of quality control measures to ensure the effectiveness and reliability of infant neurodevelopment assessment. We video-filmed the neurodevelopment assessment process with the informed consent of the guardian to judge each assessment of physician's operational standardization and scoring reasonableness. Every month, 5% of the assessment videos were selected, and pediatric specialists judged the consistency among the assessing physicians. Based on the quality control results, the assessing physicians were retrained on the issues to ensure the quality of neurodevelopmental assessment to reduce the subjective bias of the assessing physicians ^[ 22] ^.

### Covariates

We obtained potential covariables through structured questionnaires and electronic medical records. The information on study center, maternal birth date, maternal pre-pregnancy body mass index (BMI), and maternal education was collected at recruitment through face-to-face interviews during the first trimester of pregnancy. Based on the self-reported maternal height and pre-pregnancy weight, the maternal pre-pregnancy BMI was calculated. We also obtained information on the mode of conception, parity, plurality, maternal diabetes in pregnancy, date of delivery, gestational week at delivery, and infant sex from electronic medical records. The maternal age at delivery was calculated according to the maternal birth date and the date of delivery. The examination age of infants and duration of breastfeeding were collected at the one-year follow-up of infants.

### Statistical analysis

The *χ* ^2^ test and Student's *t*-test were used to compare the distributions of neurodevelopmental outcomes in infants of mothers with and without HDP. Maternal HDP exposure included non-HDP (as reference), chronic hypertension, gestational hypertension, and preeclampsia. We assessed the associations between maternal HDP and Bayley-Ⅲ Screening Test results separately for singleton and twin pregnancies, respectively. General linear regression models and Poisson regression models were used to estimate the associations between maternal HDP and infant neurodevelopment at one year old. Considering the non-independence of twins, the analyses were fitted by a linear mixed model. We developed a directed acyclic graph ( ***Supplementary Fig. 1 ***, available online) to document our assumptions about the associations among covariates, exposure, and outcome. Analyses were adjusted for maternal age at delivery (continuous), maternal pre-pregnancy BMI (continuous), parity (nulliparous or multiparous), study center (Nanjing or Suzhou), maternal education (< 12 or ≥ 12 years), mode of conception (ART or spontaneous), infant sex (boy or girl), duration of breastfeeding (< 6 or 6–12 months), and infant age at examination (continuous).

Further, the stratified and sensitivity analyses were performed for mother-infant pairs with singleton pregnancies. As gestational age may be a variable in the causal pathway ^[ 23] ^, the associations between HDP and infant neurodevelopment were stratified by preterm birth (PTB, no or yes). In addition, the study population included spontaneous- and ART-conceived families. To verify the consistency of the results, we conducted stratified analyses by mode of conception (spontaneous or ART). Finally, we excluded the infants born to mothers with diabetes in pregnancy for sensitivity analysis to assess whether the potential association could be attributed to the confounding by diabetes in pregnancy. To further control for confounding variables, we used the propensity score matching method for sensitivity analysis, using the nearest neighbor matching method, selecting no-substitution 1∶4 matching based on maternal age at delivery, maternal pre-pregnancy BMI, parity, study center, maternal education, mode of conception, diabetes in pregnancy, plurality, infant sex, duration of breastfeeding, and infant age at examination to calculate the propensity score for each offspring, matching the individual with the closest propensity score between the HDP and non-HDP groups.

Previous studies showed the incidence rate of neurodevelopment delay was 4.5% to 12.4% ^[ 12, 17, 24] ^. As a result, we set the incidence of neurodevelopment delay to 4.5% in the present study. According to the previous studies, the odds ratios of HDP exposure on neurodevelopment delay outcomes ranged from 1.80 to 2.86 ^[ 12, 17, 24] ^, and we set the odds ratio to 1.80. The alpha was set to 0.05 and the power to 0.80. The estimated sample size was 1646, and we included 2837 women in the present study. As a result, there was sufficient power to determine the real association between HDP and infant neurodevelopment.

Information on missing data is outlined in ***Table 1***, and missing data on covariates were coded as a missing indicator in multivariable regression models. All analyses were conducted in R (v4.1.0) (The Comprehensive R Archive Network, http://cran.r-project.org). Two-sided *P* < 0.05 was considered statistically significant.

**Table 1:** Basic characteristics of the study population

Characteristics	Total	Singleton	Twins
Maternal characteristics ( *n*)	2837	2576	261
Mode of conception [ *n* (%)]			
Spontaneous	1587 (55.9)	1579 (61.3)	8 (3.1)
ART	1250 (44.1)	997 (38.7)	253 (96.9)
Center [ *n* (%)]			
Nanjing	1631 (57.5)	1497 (58.1)	134 (51.3)
Suzhou	1206 (42.5)	1079 (41.9)	127 (48.7)
Maternal age (years, mean±SD)	31.17±3.81	31.16±3.88	31.20±3.09
Pre-pregnancy BMI [kg/m ^2^, *n* (%)]			
<18.5 kg/m ^2^	287 (10.1)	269 (10.4)	18 (6.9)
[18.5, 24.0) kg/m ^2^	1939 (68.4)	1763 (68.5)	176 (67.4)
≥24.0 kg/m ^2^	610 (21.5)	543 (21.1)	67 (25.7)
Missing	1 (0.0)	1 (0.0)	0
Maternal education [ *n* (%)]			
<12 years	459 (16.2)	396 (15.4)	63 (24.1)
≥12 years	2378 (83.8)	2180 (84.6)	198 (75.9)
Parity [ *n* (%)]			
Nulliparous	2243 (79.1)	2008 (77.9)	235 (90.0)
Multiparous	550 (19.4)	532 (20.7)	18 (6.9)
Missing	44 (1.5)	36 (1.4)	8 (3.1)
Diabetes in pregnancy [ *n* (%)]			
No	1874 (66.0)	1714 (66.5)	160 (61.3)
Yes	961 (33.9)	860 (33.4)	101 (38.7)
Missing	2 (0.1)	2 (0.1)	0
Gestational age (weeks, mean±SD)	39.16±1.62	39.44±1.34	36.43±1.63
PTB [ *n* (%)]			
No	2 598 (91.6)	2 463 (95.6)	135 (51.7)
Yes	239 (8.4)	113 (4.4)	126 (48.3)
HDP [ *n* (%)]	220 (7.7)	162 (6.3)	58 (22.2)
Chronic hypertension	46 (1.6)	41 (1.6)	5 (1.9)
Gestational hypertension	71 (2.5)	55 (2.1)	16 (6.1)
Preeclampsia	103 (3.6)	66 (2.6)	37 (14.2)
Infant characteristics ( *n*)	3 083	2 576	507
Birth weight (g, mean±SD)	3251.98±538.86	3387.28±446.95	2565.27±431.20
Infant sex [ *n* (%)]			
Boys	1 629 (52.8)	1 367 (53.1)	262 (51.7)
Girls	1454 (47.2)	1209 (46.9)	245 (48.3)
Duration of breastfeeding [ *n* (%)]			
<6 months	630 (20.4)	424 (16.5)	206 (40.6)
6–12 months	2445 (79.3)	2146 (83.3)	299 (59.0)
Missing	8 (0.3)	6 (0.2)	2 (0.4)
Examination age of infant (days, mean±SD)	365.04±7.82	364.91±7.82	365.70±7.82
Abbreviations: ART, assisted reproductive technology; SD, standard deviation; BMI, body mass index; PTB, preterm birth; HDP, hypertensive disorders in pregnancy.			

## Results

### Characteristics of the participants

A total of 2837 families were included in the present study, including 2576 singleton pregnancies and 261 twin pregnancies. The basic characteristics of the study participants are summarized in ***Table 1***. Of the 2837 mothers, 220 (7.7%) of those had HDP, including 46 (1.6%) with chronic hypertension, 71 (2.5%) with gestational hypertension, and 103 (3.6%) with preeclampsia. The mean age of mothers at delivery was 31 years old. Nearly 80% of mothers were nulliparous and had more than 12 years of education. Approximately 21.5% of mothers were overweight or obese before pregnancy, 33.9% of mothers had diabetes in pregnancy, including pre-pregnancy diabetes and gestational diabetes mellitus, and 8.4% of mothers gave birth to a premature infant. Of the 3083 infants, the mean birth weight was 3251.98 g, 47.2% of infants were girls, and 79.3% of infants breastfed for more than six months. Among twin pregnancies, the incidence of HDP was 22.2%, including five (1.9%) with chronic hypertension, 16 (6.1%) with gestational hypertension, and 37 (14.2%) with preeclampsia. Almost all (96.9%) twin pregnancies were conceived through ART. Among singleton pregnancies, the incidence of HDP was 6.3%, including 41 (1.6%) with chronic hypertension, 55 (2.1%) with gestational hypertension, and 66 (2.6%) with preeclampsia.

***Supplementary Table 2*** (available online) shows the distribution of infant neurodevelopment. Among singleton pregnancies, compared with those infants born to mothers with non-HDP exposure, infants born to mothers with HDP had lower scores in expressive communication (11.80 *vs.* 12.12, *P* = 0.035) and gross motor (14.29 *vs.* 14.66, *P* = 0.005). Moreover, infants born to mothers with HDP had a higher prevalence of "non-optimal" development in the domain of gross motor (12.3% *vs.* 8.5 %), but the difference was not statistically significant ( *P* = 0.129). Among twin pregnancies, compared with those infants born to mothers with non-HDP exposure, infants born to mothers with HDP had lower scores in receptive communication (10.03 *vs.* 10.53, *P* = 0.018) and gross motor (13.91 *vs.* 14.35, *P* = 0.011), but had a higher prevalence of "non-optimal" development in gross motor (21.4% *vs.* 10.4%, *P* = 0.003).

### Associations between exposure to HDP and infant neurodevelopment score

The associations between exposure to HDP and infant neurodevelopment scores are shown in ***Table 2***. In singletons, after adjusting for confounding factors, we found that infants born to mothers with HDP had a lower score in gross motor ( *β*, −0.32; 95% confidence interval [CI], −0.59–−0.05), and infants born to mothers with chronic hypertension also had a lower gross motor score ( *β*, −0.67; 95% CI, −1.19–−0.15). In twin pregnancies, after adjustment for confounding factors, infants born to mothers with HDP had lower scores in cognition ( *β*, −0.49; 95% CI, −0.96–−0.01), receptive communication ( *β*, −0.55; 95% CI, −1.03–−0.06), and gross motor ( *β*, −0.44; 95% CI, −0.86–−0.03); infants born to mothers with chronic hypertension had lower scores in cognition ( *β*, −1.52; 95% CI, −2.97–−0.06), and receptive communication ( *β*, −2.03; 95% CI, −3.50–−0.56); and infants born to mothers with gestational hypertension also had lower scores in cognition ( *β*, −1.04; 95% CI, −1.85–−0.23), receptive communication ( *β*, −0.89; 95% CI, −1.71–−0.07), and expressive communication ( *β*, −1.02; 95% CI, −1.89–−0.15).

**Table 2:** Associations between exposure to HDP and infant neurodevelopment scores at one year of age

Characters	Singleton ( *n*=2576)					Twins ( *n*=507)			
	*N*	Score, mean±SD	*β* (95% CI) ^a^	*P*		*N*	Score, mean±SD	*β* (95% CI) ^a^	*P*
Cognition	2576					507			
Non-HDP	2414	15.74±1.88	Ref			395	15.02±1.99	Ref	
HDP	162	15.56±1.93	–0.09 (–0.40, 0.23)	0.589		112	14.63±1.92	**–0.49 (–0.96, –0.01)**	**0.046**
Chronic hypertension	41	15.78±1.86	0.20 (–0.40, 0.80)	0.511		9	13.56±1.24	**–1.52 (–2.97, –0.06)**	**0.043**
Gestational hypertension/ Preeclampsia	121	15.49±1.96	–0.18 (–0.54, 0.17)	0.32		103	14.73±1.95	–0.39 (–0.88, 0.10)	0.123
Gestational hypertension	55	15.25±2.20	–0.37 (–0.89, 0.14)	0.156		31	13.90±1.90	**–1.04 (–1.85, –0.23)**	**0.013**
Preeclampsia	66	15.68±1.73	–0.02 (–0.49, 0.45)	0.933		72	15.08±1.87	–0.09 (–0.66, 0.49)	0.764
Receptive communication	2576					507			
Non-HDP	2414	11.23±1.96	Ref			395	10.53±1.95	Ref	
HDP	162	11.13±1.78	0.06 (–0.26, 0.38)	0.702		112	10.03±2.15	**–0.55 (–1.03, –0.06)**	**0.028**
Chronic hypertension	41	11.15±1.67	0.09 (–0.52, 0.71)	0.764		9	8.44±1.59	**–2.03 (–3.50, –0.56)**	**0.007**
Gestational hypertension/ Preeclampsia	121	11.12±1.82	0.05 (–0.31, 0.42)	0.779		103	10.17±2.15	–0.41 (–0.90, 0.09)	0.112
Gestational hypertension	55	10.96±2.06	0.00 (–0.53, 0.52)	0.987		31	9.48±2.16	**–0.89 (–1.71, –0.07)**	**0.035**
Preeclampsia	66	11.26±1.59	0.10 (–0.38, 0.58)	0.688		72	10.46±2.09	–0.18 (–0.76, 0.40)	0.543
Expressive communication	2576					507			
Non-HDP	2414	12.12±1.89	Ref			395	11.57±2.05	Ref	
HDP	162	11.80±1.90	–0.20 (–0.51, 0.12)	0.215		112	11.24±2.33	–0.40 (–0.91, 0.11)	0.122
Chronic hypertension	41	11.59±2.31	–0.30 (–0.90, 0.31)	0.337		9	10.62±3.16	–1.10 (–2.72, 0.52)	0.186
Gestational hypertension/ Preeclampsia	121	11.87±1.75	–0.17 (–0.52, 0.19)	0.36		103	11.29±2.27	–0.34 (–0.87, 0.19)	0.206
Gestational hypertension	55	11.85±1.98	–0.10 (–0.62, 0.42)	0.709		31	10.35±2.59	**–1.02 (–1.89, –0.15)**	**0.022**
Preeclampsia	66	11.88±1.54	–0.22 (–0.70, 0.25)	0.356		72	11.69±2.00	–0.03 (–0.64, 0.59)	0.932
Fine motor	2576					507			
Non-HDP	2414	13.03±1.51	Ref			395	12.50±1.41	Ref	
HDP	162	12.88±1.52	–0.05 (–0.30, 0.20)	0.686		112	12.50±1.37	–0.04 (–0.35, 0.28)	0.82
Chronic hypertension	41	12.63±1.34	–0.28 (–0.76, 0.20)	0.249		9	12.22±1.30	–0.24 (–1.21, 0.74)	0.632
Gestational hypertension/ Preeclampsia	121	12.97±1.58	0.02 (–0.26, 0.31)	0.868		103	12.52±1.38	–0.02 (–0.35, 0.31)	0.916
Gestational hypertension	55	12.96±1.43	0.09 (–0.32, 0.50)	0.661		31	12.16±1.53	–0.24 (–0.78, 0.30)	0.387
Preeclampsia	66	12.97 ± 1.70	–0.03 (–0.41, 0.34)	0.867		72	12.68±1.29	0.09 (–0.30, 0.47)	0.664
Gross motor	2576					507			
Non-HDP	2414	14.66±1.63	Ref			395	14.35±1.61	Ref	
HDP	162	14.29±1.61	**–0.32 (–0.59, –0.05)**	**0.019**		112	13.91±1.65	**–0.44 (–0.86, –0.03)**	**0.037**
Chronic hypertension	41	13.98±1.56	**–0.67 (–1.19, –0.15)**	**0.012**		9	13.33±1.50	–0.82 (–2.08, 0.44)	0.204
Gestational hypertension/ Preeclampsia	121	14.40±1.62	–0.21 (–0.52, 0.10)	0.18		103	13.96±1.66	–0.41 (–0.84, 0.02)	0.065
Gestational hypertension	55	14.25±1.43	–0.27 (–0.72, 0.17)	0.228		31	13.65±1.50	–0.69 (–1.41, 0.02)	0.058
Preeclampsia	66	14.52±1.76	–0.16 (–0.56, 0.25)	0.453		72	14.10±1.72	–0.28 (–0.78, 0.23)	0.286
Data are presented as *β* (95% CI). The employed statistical analysis methodology encompassed general linear regression models. In the case of twins, the analyses were fitted by linear mixed model. ^a^Adjusted for mode of conception, maternal age at delivery, pre-pregnancy BMI, parity, infant sex, duration of breastfeeding, examination age of infant, maternal education, and study center. Bold font indicates *P* < 0.05. Abbreviations: HDP, hypertensive disorders in pregnancy; CI, confidence interval; SD, standard deviation.									

### Relative risk for exposure to HDP in "non-optimal" develpment in infants at one year of age

Relative risk for exposure to HDP in "non-optimal" develpment in infants at one year of age is shown in ***Table 3***. In singletons, we found that maternal chronic hypertension was positively associated with "non-optimal" development in gross motor (risk ratio, [RR], 2.21; 95% CI, 1.02–4.79). Among twin pregnancies, maternal HDP was positively associated with "non-optimal" development in gross motor (RR, 2.12; 95% CI, 1.16–3.88); maternal chronic hypertension exposure was positively associated with "non-optimal" infant development in cognition and receptive communication after adjusting for confounding factors (cognition: RR, 2.84; 95% CI, 1.11–7.25; receptive communication: RR, 2.48; 95% CI, 1.06–5.81); infants born to mothers with gestational hypertension increased risks of "non-optimal" development in cognition (RR, 1.87; 95% CI, 1.01–3.48), expressive communication (RR, 3.25; 95% CI, 1.29–8.23), and gross motor (RR, 2.77; 95% CI, 1.13–6.79). In addition, we found that infants born to mothers with gestational hypertension or preeclampsia were at 2.19-fold (95% CI, 1.18–4.10) risk of "non-optimal" in the gross motor development than those mothers non-exposed to HDP.

**Table 3:** Relative risk for exposure to HDP in "non-optimal" develpment in infants at one year of age in linear mixed models

Characters	Singleton ( *n*=2576)				Twins ( *n*=507)		
	*no*/ *No* (%)	RR (95% CI) ^a^	*P*		*no*/ *No* (%)	RR (95% CI) ^a^	*P*
Cognition							
Non-HDP	282/2414 (11.7)	Ref			79/395 (20.0)	Ref	
HDP	24/162 (14.8)	1.24 (0.81, 1.91)	0.324		32/112 (28.6)	1.44 (0.94, 2.19)	0.092
Chronic hypertension	5/41 (12.2)	0.97 (0.40, 2.38)	0.949		5/9 (55.6)	**2.84 (1.11, 7.25)**	**0.029**
Gestational hypertension/Preeclampsia	19/121 (15.7)	1.34 (0.83, 2.16)	0.230		27/103 (26.2)	1.31 (0.83, 2.05)	0.240
Gestational hypertension	11/55 (20.0)	1.65 (0.89, 3.04)	0.111		12/31 (38.7)	**1.87 (1.01, 3.48)**	**0.047**
Preeclampsia	8/66 (12.1)	1.07 (0.52, 2.17)	0.860		15/72 (20.8)	1.05 (0.60, 1.85)	0.865
Receptive communication							
Non-HDP	408/2414 (16.9)	Ref			109/395 (27.6)	Ref	
HDP	22/162 (13.6)	0.66 (0.42, 1.06)	0.088		40/112 (35.7)	1.28 (0.88, 1.85)	0.199
Chronic hypertension	4/41 (9.8)	0.44 (0.14, 1.37)	0.157		6/9 (66.7)	**2.48 (1.06, 5.81)**	**0.036**
Gestational hypertension/Preeclampsia	18/121 (14.9)	0.73 (0.44, 1.22)	0.232		34/103 (33.0)	1.17 (0.79, 1.74)	0.440
Gestational hypertension	13/55 (23.6)	1.06 (0.58, 1.94)	0.851		13/31 (41.9)	1.42 (0.79, 2.55)	0.245
Preeclampsia	5/66 (7.6)	0.44 (0.18, 1.06)	0.068		21/72 (29.2)	1.05 (0.65, 1.70)	0.831
Expressive communication							
Non-HDP	121/2414 (5.0)	Ref			32/395 (8.1)	Ref	
HDP	9/162 (5.6)	1.02 (0.46, 2.22)	0.968		16/112 (14.3)	1.61 (0.77, 3.34)	0.203
Chronic hypertension	4/41 (9.8)	1.83 (0.57, 5.88)	0.312		1/9 (11.1)	2.23 (0.24, 20.87)	0.483
Gestational hypertension/Preeclampsia	5/121 (4.1)	0.76 (0.28, 2.10)	0.602		15/103 (14.6)	1.53 (0.73, 3.19)	0.256
Gestational hypertension	4/55 (7.3)	1.25 (0.39, 3.97)	0.711		9/31 (29.0)	**3.25 (1.29, 8.23)**	**0.013**
Preeclampsia	1/66 (1.5)	0.35 (0.05, 2.55)	0.303		6/72 (8.3)	0.88 (0.32, 2.39)	0.799
Fine motor							
Non-HDP	78/2414 (3.2)	Ref			23/395 (5.8)	Ref	
HDP	5/162 (3.1)	1.09 (0.43, 2.75)	0.858		8/112 (7.1)	1.09 (0.48, 2.49)	0.837
Chronic hypertension	2/41 (4.9)	1.83 (0.44, 7.64)	0.406		1/9 (11.1)	3.53 (0.43, 29.03)	0.241
Gestational hypertension/Preeclampsia	3/121 (2.5)	0.86 (0.27, 2.77)	0.798		7/103 (6.8)	0.98 (0.41, 2.34)	0.968
Gestational hypertension	1/55 (1.8)	0.57 (0.08, 4.15)	0.579		3/31 (9.7)	1.38 (0.41, 4.69)	0.604
Preeclampsia	2/66 (3.0)	1.15 (0.28, 4.75)	0.849		4/72 (5.6)	0.80 (0.27, 2.38)	0.691
Gross motor							
Non-HDP	206/2414 (8.5)	Ref			41/395 (10.4)	Ref	
HDP	20/162 (12.3)	1.42 (0.88, 2.29)	0.147		24/112 (21.4)	**2.12 (1.16, 3.88)**	**0.015**
Chronic hypertension	7/41 (17.1)	**2.21 (1.02, 4.79)**	**0.044**		2/9 (22.2)	1.51 (0.27, 8.45)	0.642
Gestational hypertension/Preeclampsia	13/121 (10.7)	1.20 (0.67, 2.12)	0.539		22/103 (21.4)	**2.19 (1.18, 4.10)**	**0.014**
Gestational hypertension	5/55 (9.1)	1.04 (0.42, 2.54)	0.933		8/31 (25.8)	**2.77 (1.13, 6.79)**	**0.027**
Preeclampsia	8/66 (12.1)	1.32 (0.65, 2.71)	0.445		14/72 (19.4)	1.90 (0.93, 3.91)	0.080
Data are presented as RR (95% CI). The employed statistical analysis methodology comprised Poisson regression models. In the case of twins, the analyses were fitted by linear mixed model. *no*/ *No*, no., number of non-optimal; No., number of participants. ^a^Adjusted for a mode of conception, maternal age at delivery, pre-pregnancy BMI, parity, infant sex, duration of breastfeeding, maternal education, and study center. Abbreviations: HDP, hypertensive disorders in pregnancy; CI, confidence interval; RR, risk ratio.							

### Stratified analyses of associations between exposure to maternal HDP and infant neurodevelopment scores among singleton pregnancies

**Figure 2:**
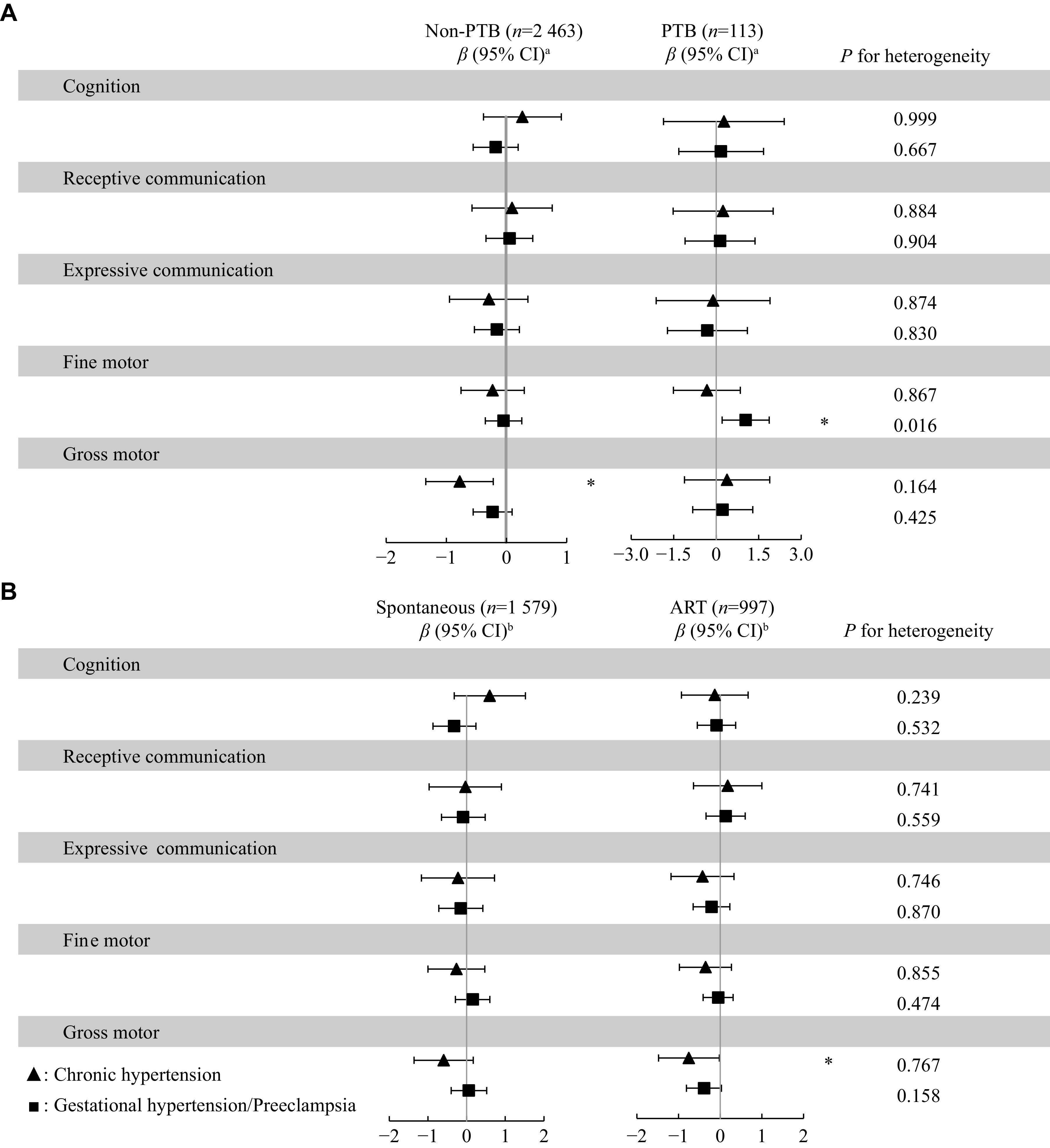
Stratified analyses of associations between exposure to maternal HDP and infant neurodevelopment scores among singleton pregnancies.

**Figure 3:**
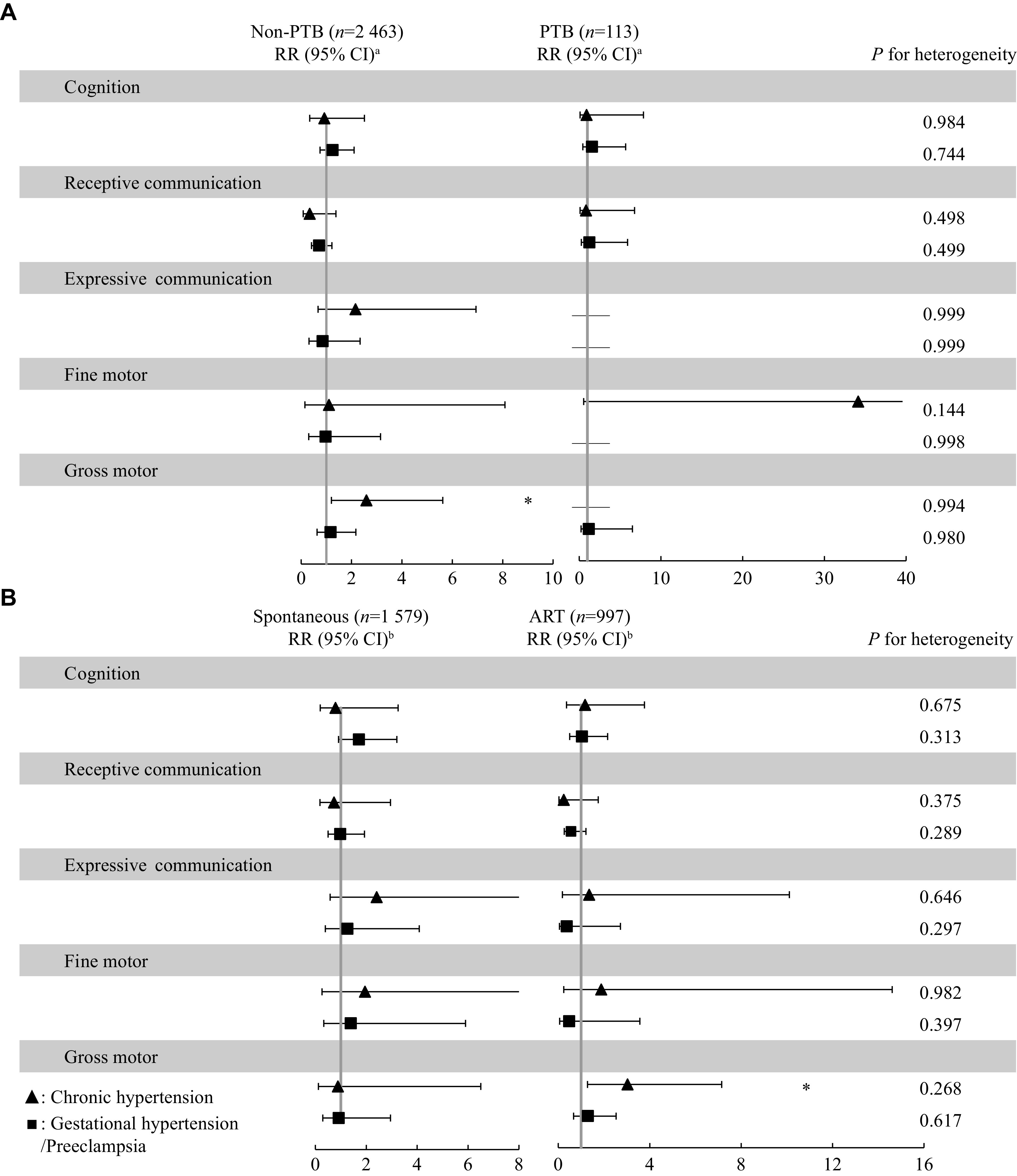
Stratified analyses of relative risk for exposure to HDP in "non-optimal" development in infants at one year of age among singleton pregnancies.

Maternal chronic hypertension was significantly associated with lower gross motor scores among 2463 term-born singleton infants ( *β*, −0.76; 95% CI, −1.32–−0.21), but not statistically significant among 113 preterm-born singleton infants. However, the heterogeneity test was not significant ( *P* for heterogeneity = 0.164) ( ***Fig. 2A*** and ***Supplementary Table 3*** [available online]). Moreover, the heterogeneity between term-born and preterm-born singleton infants was statistically significant for the association of gestational hypertension or preeclampsia with fine motor score ( *P* for heterogeneity = 0.016). Maternal exposure to gestational hypertension or preeclampsia was significantly associated with the increased score on the fine motor ( *β*, 1.04; 95% CI, 0.21–1.87) among preterm-born singleton infants, but not statistically significant in term-born singleton infants ( ***Fig. 2A*** and ***Supplementary Table 3 ***). In addition, we found that infants born to mothers with chronic hypertension had a higher risk of "non-optimal" development of the gross motor in term-born infants (RR, 2.59; 95% CI, 1.20–5.62), but none of the mothers of preterm infants had chronic hypertension ( ***Fig. 3A*** and ***Supplementary Table 4*** [available online]). In the stratified analysis by mode of conception, a significant negative association of maternal chronic hypertension with infant gross motor score was observed in ART-conceived singleton infants ( *β*, −0.75; 95% CI, −1.48–−0.02). The negative association was shown among spontaneous-conceived singleton infants but not statistically significant ( *β*, −0.59; 95% CI, −1.36–0.17, *P* for heterogeneity = 0.767) ( ***Fig. 2B*** and ***Supplementary Table 3 ***). Finally, we found that infants born to mothers with chronic hypertension had a higher risk of "non-optimal" development of the gross motor in ART-conceived infants (RR, 3.03; 95% CI, 1.28–7.15), but not among spontaneous-conceived infants (RR, 0.89; 95% CI, 0.12–6.50); however, the heterogeneity test was not significant ( *P* for heterogeneity = 0.268) ( ***Fig. 3B*** and ***Supplementary Table 4 ***)

### Sensitivity analysis

When restricting the study population to infants born to mothers without diabetes in pregnancy, we still found that infants of mothers with chronic hypertension had a lower gross motor score ( *β*, −1.11; 95% CI, −1.84–−0.39), and no significant association was found between maternal chronic hypertension and risk of "non-optimal" gross motor development in infants at one year of age (RR = 1.86, 95% CI: 0.58, 6.01) ( ***Supplementary Tables 5*** and ***6 ***, available online). In addition, the results of propensity score matching were consistent with the main analysis that maternal HDP had a negative effect on neurodevelopment of infants ( ***Supplementary Tables 7*** and ***8 ***, available online).

## Discussion

The present prospective cohort study was based on Jiangsu Birth Cohort to investigate the associations between maternal HDP and infant neurodevelopment at one year old. Among singleton pregnancies, we found that maternal chronic hypertension had a negative effect on gross motor development. Among twin pregnancies, we found that maternal HDP was significantly associated with impaired development in cognitive and receptive communication, expressive communication, and gross motor domains. These findings highlight the need to strengthen pediatric monitoring of infants, whose mothers have been exposed to HDP, to provide early interventions that may help improve neurodevelopmental outcomes, especially for multiple pregnancies.

In singleton pregnancies, we found that infants of mothers with HDP had poorer developmental outcomes on the gross motor. One previous study also found a negative effect of maternal chronic hypertension on infant neurodevelopment. For example, a prospective cohort study in Wuhan that included 4031 singleton live births found that maternal chronic hypertension increased the risk of neurodevelopment impairment in fine motor, adaptability, language and social behavior of infants at six months ^[ 12] ^. However, several studies reported a lack of significant association between maternal chronic hypertension and offspring neurodevelopment. For example, the Northern Finland Birth Cohort followed up 8847 singleton children for up to 11.5 years and found no significant association between chronic hypertension and mild cognitive impairment in the offspring ^[ 17] ^. There was also a prospective cohort study from Western Australia that did not find a significant association between chronic hypertension and motor development in the offspring during adolescence ^[ 25] ^. It is noteworthy to mention that, the neurodevelopmental domains of the offspring affected by chronic hypertension in that Western Australian study were not consistent with ours in the present study, probably due to the inconsistency of the development scale and the ages assessed in the different studies.

The present study also investigated the effect of gestational hypertension or preeclampsia on infant neurodevelopment, but did not find a significant association of gestational hypertension or preeclampsia with infant neurodevelopment in singleton pregnancies, a finding consisted with that of two previous studies ^[ 18, 26] ^. One of those studies involved a longitudinal study cohort from Australia, which utilized the Australian version of the Canadian Early Development Instrument to measure cognitive skills, language, communication skills, and motor development of offspring at the age of five years, and did not find a significant association between maternal gestational hypertension or preeclampsia and infants cognitive, language, communication skills, and motor development ^[ 26] ^. Similarly, another study, derived from a Canadian community-based population cohort, employed the Ages and Stages Questionnaires to assess infant neurodevelopment of three-year-old, and did not find maternal gestational hypertension or preeclampsia to be associated with an increased risk of motor and cognitive delays ^[ 18] ^. However, several other studies also found negative effects of gestational hypertension or preeclampsia on cognitive, language and motor development in the offspring. For example, in 2011, one study found that infants born to mothers with severe HDP showed more cognitive problems at the age of 4.5-year-old than those with non-HDP exposure ^[ 27] ^; another prospective cohort study in Western Australia, using the Peabody Picture Vocabulary Test-Revised to assess offspring neurodevelopment, showed that maternal gestational hypertension or preeclampsia was significantly associated with reduced language ability scores in offspring at the age of 10 years; and their further analyses found that maternal gestational hypertension reduced language scores by 1.71 points and that preeclampsia reduced scores by 3.53 points, but the association between preeclampsia and offspring language development was not statistically significant ^[ 18] ^. Nevertheless, two studies revealed that maternal HDP, preeclampsia in particular, was associated with motor development ^[ 25, 28] ^, but maternal chronic hypertension had no significant effect on the development of gross motor ^[ 28] ^. The reasons for these inconsistencies may be that the time for evaluating neurodevelopment was different, ranging between three and 10 years, because a longer follow-up may be more likely to observe a significant association between maternal HDP and infant development problems ^[ 18] ^. Another possible reason is the difference in neurodevelopmental assessment scales used in these studies.

We further found that maternal gestational hypertension or preeclampsia had a positive effect on the fine motor of preterm singletons, but the association was not statistically significant in term infants. The result was consistent with that of a study in which infants of mothers with preeclampsia had a higher Psychomotor Developmental Index score in low-birth-weight infants ^[ 14] ^. It is noteworthy to mention that in the ART-conceived singletons, maternal chronic hypertension increased the risk of "non-optimal" development in gross motor, but in the spontaneous-conceived infants, the association was not statistically significant; however, the subsequent heterogeneity test was not significant. This may be due to a higher incidence of HDP in the ART-conceived mothers.

Among twin pregnancies, we found that infants of mothers with HDP had poorer developmental outcomes in the cognitive, language, and gross motor. Twin pregnancy is a well-known risk factor for later neurodevelopment ^[ 29] ^. We found that maternal HDP significantly increased this risk. To date, the association between maternal HDP and infant neurodevelopment has not been investigated in twin pregnancies. The present study provided evidence for the association between maternal HDP and infant neurodevelopment among twin pregnancies, but more studies are needed to replicate our findings.

Maternal HDP may affect the developing brain through the following mechanisms. First, the HDP-related constriction of blood vessels may lead to hypoxia in the placental environment ^[ 30] ^, because animal models showed that hypoxia damage might lead to permanent changes in brain structure ^[ 7] ^. The poor motor performance of offspring may be due to the interruption of the development and function of the cerebellum and related neural pathways caused by placental dysfunction ^[ 25] ^. Cerebellar function is implied in the coordination of movements as well as in the establishment and execution of motor skills ^[ 25, 31] ^. In addition, inflammation is also a potential mechanism. It has been found that the levels of proinflammatory cytokines in the plasma of women with HDP increased cytokine-mediated inflammation, which may affect the fetal brain by directly damaging neurons or their surrounding tissues ^[ 32– 33] ^. These mechanisms warrant additional investigations to identify therapeutic targets to prevent neurodevelopmental disorders in the infant of mothers with HDP.

The present study has some advantages. The main advantage was the prospective cohort study design that provided accurate exposure and covariate data. Additionally, the effects of different types of HDP, such as chronic hypertension, gestational hypertension, and preeclampsia, on neurodevelopment were compared. Finally, we used the Bayley-Ⅲ Screening Test to assess the neurodevelopment of infants at one year old, which is one of the internationally used developmental scales with a high reliability and validity ^[ 21] ^. Besides, the neurodevelopmental assessment was completed by professionally trained physicians, who were not aware of the mother's HDP diagnosis at the time of the assessment, to keep the results objective and unbiased.

The limitations of the present study should also be noted. First, data on the onset time and severity of HDP were unavailable and precluded us from elucidating the associated effects of early versus late-onset, or mild versus severe HDP on neurodevelopmental outcomes. Second, although the present study had a good study design and strict quality control measures, we were still unable to fully consider all confounding factors, including detailed information about maternal antipsychotic medication, parental intellectual development, and postnatal family education. Third, our study investigated the associations between HDP and the neurodevelopment of infants at one year of age, but most of the previous studies have a longer follow-up time, which was likely to cause our results not directly comparable. Furthermore, maternal HDP was determined based on childbirth records in the medical information system, but the postpartum HDP data were not considered, and thus potential misclassification of HDP remains. Third, the study population included the ART-conceived and spontaneous-conceived infants, and the extrapolation of the study to the general population was limited. Finally, although our sample size was quite large, when HDP was subdivided into subtypes, the sample size was too small and the statistical power decreased. Studies with larger sample sizes are still needed to further explore the effects of different HDP types on infant neurodevelopment.

In summary, among singleton pregnancies, maternal HDP had a negative impact on the gross motor development of infants at one year old. Among twin pregnancies, maternal HDP also had negative impacts on cognition, receptive communication, expressive communication, and gross motor development of infants at one year old. Finally, infants born to mothers with HDP had poorer neurodevelopment outcomes at one year old, especially for the twin pregnancies. These findings emphasize the necessity to continue the follow-up of these infants and carry out early intervention to improve neurodevelopmental outcomes in the future.
